# A novel function of RIP1 in postnatal development and immune homeostasis by protecting against RIP3-dependent necroptosis and FADD-mediated apoptosis

**DOI:** 10.3389/fcell.2015.00012

**Published:** 2015-02-25

**Authors:** John P. Dowling, Anirudh Nair, Jianke Zhang

**Affiliations:** Department of Microbiology and Immunology, Sidney Kimmel Cancer Center, Sidney Kimmel Medical College, Thomas Jefferson UniversityPhiladelphia, PA, USA

**Keywords:** mouse development, apoptosis, necroptosis, programmed cell death (PCD), immune homeostasis

## Abstract

RIP1 is an adaptor kinase originally identified as being able to associate with TNFR1 and Fas, and is later shown to be involved in signaling induced by TLRs. Major signaling pathways regulated by RIP1 include necroptosis, apoptosis, and pro-survival/inflammation NF-κB activation. Previous studies show that RIP1 deficiency has no effect on mouse embryogenesis, but blocks postnatal development. This phenotype could not readily be explained, since mice lacking TNFR1, Fas, or TLRs show no apparent developmental defect. Certain types of RIP1-deficient cells are hypersensitive to TNF-induced apoptosis. However, in our previous study, deletion of the apoptotic adaptor protein, FADD, provides marginal improvement of postnatal development of *rip1*^−/−^ mice. Remarkably, the current data shows that haploid insufficiency of RIP3, a known mediator of necroptosis, allowed survival of *rip1*^−/−^*fadd*^−/−^ mice beyond weaning age, although the resulting *rip1^−/−^fadd^−/−^ rip3^+/−^* mice were significant smaller in size and weight. Moreover, complete absence of RIP3 further improved postnatal development of the resulting *rip1*^−/−^*fadd*^−/−^*rip3*^−/−^ mice, which display normal size and weight. In such triple knockout (TKO) mice, lymphocytes underwent normal development, but progressively accumulated as mice age. This lymphoproliferative (*lpr*) disease in TKO mice is, however, less severe than that of *fadd^−/−^rip3*^−/−^ double knockout mice. In total, the data show that the postnatal developmental defect in *rip1*^−/−^ mice is due in part to FADD-mediated apoptosis as well as RIP3-dependent necroptosis. Moreover, the function of RIP1 contributes to development of *lpr* diseases.

## Introduction

Apoptosis and necroptosis are programmed cell death (PCD) pathways that play an essential role during animal development and immune responses (Moriwaki and Chan, 2013; Walsh, 2014). Although these two PCD pathways have distinct mechanisms and outcomes, they are intricately connected and, therefore, must be tightly regulated. PCD can be initiated through the death receptors (DRs) like Fas and tumor necrosis factor receptor-1 (TNFR1) (Ashkenazi and Dixit, 1998). Apoptotic death induced by DRs is mediated by the Fas-associated death domain (FADD or MORT1) adaptor protein (Boldin et al., 1995; Chinnaiyan et al., 1995; Zhang and Winoto, 1996). FADD binds to and activates the apical Caspase 8, which then cleaves and activates the effector Caspases, and triggers the apoptotic program (Boldin et al., 1996; Muzio et al., 1996). When apoptotic pathway is blocked, such as through viral defense mechanisms or Caspase inhibitory conditions, DRs signaling could lead to necroptosis which is mediated through the receptor interacting protein (RIP)1-RIP3-mixed lineage kinase domain-like (MLKL) protein axis (Holler et al., 2000; Degterev and Yuan, 2008; Cho et al., 2009; Sun et al., 2012; Zhao et al., 2012).

One striking example of the importance of these PCD pathways in immune homeostasis is illustrated by a mutant mouse strain with lymphoproliferative (*lpr*) disease caused by mutations in the *fas* gene (Strasser et al., 2009). In *lpr* mice, lymphocytes are unable to undergo Fas-mediated cell death, leading to accumulation of a unique subset of T cells that lose expression of CD4 and CD8 and gain expression of B220, a traditional B cell marker (Strasser et al., 2009). Moreover, in certain genetic backgrounds, the *lpr* Fas mutation facilitates autoimmunity including glomerulonephritis, arthritis, and autoantibody production, and diseases sharing symptoms of systemic lupus erythematosus (SLE) (Cohen and Eisenberg, 1992; Shi et al., 2002). In human patients containing mutations in the *fas* gene, a similar disease has been identified as autoimmune-lymphoproliferative symdrome (ALPS) (Fisher et al., 1995). Deletion of TNFR1 alone does not affect lymphoid homeostasis (Pfeffer et al., 1993; Rothe et al., 1993), but exacerbates the *lpr* disease of Fas mutant mice (Zhou et al., 1996).

Besides abnormality in certain immune functions, mice lacking each individual DR undergo normal embryogenesis and postnatal development in mice (Pfeffer et al., 1993; Rothe et al., 1993; Adachi et al., 1996; Diehl et al., 2004). Surprisingly, inactivation of FADD, the common signaling adaptor of the DRs, results in death around embryonic day (E)11.5 (Yeh et al., 1998; Zhang et al., 1998). It has been demonstrated that *fadd*^−/−^ embryos contain elevated levels of RIP1 and undergo massive necrosis (Zhang et al., 2011). Lack of RIP1 completely corrects the embryonic defect in *fadd*^−/−^ embryos. Moreover, RIP3 deletion prevents T cell death induced by expression of a FADD dominant-negative (DN) mutant (Lu et al., 2011). Unlike RIP1, RIP3 does not play a role in NF-κB activation (Newton et al., 2004). Deletion of RIP3 completely restores normal development of mice lacking FADD or Caspase 8 (Ch'en et al., 2011; Kaiser et al., 2011; Oberst et al., 2011), indicating that RIP1/RIP3-dependent necroptosis is unleashed in the absence of FADD or Caspase 8.

Though RIP1 was initially identified as a Fas-binding protein (Stanger et al., 1995), apoptosis through Fas is not affected in *rip1*^−/−^ cells (Ting et al., 1996; Kelliher et al., 1998). However, RIP1 deficiency leads to hypersensitivity to TNF-induced apoptosis, which has been attributed in part to a defect in NF-κB activation in certain cell types. When signaling through receptors like TNFR1 or toll like receptors (TLRs), the ubiquitination status of RIP1 can determine whether a cell lives or dies (Vucic et al., 2011; Schmukle and Walczak, 2012). For example, upon activation of TNFR1, RIP1 promotes NF-κB activation when ubiquitinated, but mediates apoptosis when deubiquitinated and associated with FADD and Caspase 8. Furthermore, if the function of FADD or Caspase 8 is interrupted, RIP1 kinase activity signals through RIP3, leading to necroptosis (Moriwaki and Chan, 2013; Vanden Berghe et al., 2014). These multiple functions of RIP1 participate in regulating inflammatory signaling and have been a target of interest for inflammatory disease (Moriwaki and Chan, 2013; Vanden Berghe et al., 2014; Walsh, 2014).

The importance of RIP1 during development is manifested in *rip1*^−/−^ mice, which die at birth with extensive cell death in the lymphoid and adipose tissues (Kelliher et al., 1998) Since without RIP1, TNFR1 initiates FADD-mediated apoptosis (Cusson et al., 2002; O'Donnell and Ting, 2011), it was surprising that deletion of FADD does not rescue *rip1*^−/−^ mice from perinatal lethality (Zhang et al., 2011). This suggests that RIP1 could play different roles during embryonic development, where it may initiate necroptosis in the absence of FADD, and in perinatal mice, where RIP1 is essential for survival. Unlike perinatal lethality in *rip1*^−/−^ or *rip1*^−/−^*fadd*^−/−^ mice, normal embryonic and postnatal development is observed in *fadd^−/−^rip3*^−/−^ mice (Dillon et al., 2012). Moreover, under certain circumstances, RIP3 is able to signal necroptosis in the absence of RIP1 (Upton et al., 2010; Moujalled et al., 2013). In this study, we performed mouse genetic studies and show that lack of RIP3 is insufficient to correct the defect in RIP1 mice. However, the resulting *rip1^−/−^rip3*^−/−^ mice could survive to adulthood if FADD is also inactivated. Therefore, our data indicate that RIP1 has a novel function, which appears to provide protection against RIP3-dependent necroptosis and FADD-mediated apoptosis during postnatal development.

## Materials and methods

### Mice

*fadd^+/−^* mice have been previously described (Zhang et al., 1998). *rip1^+/−^* mice were provided by Dr. Michelle Kelliher (Kelliher et al., 1998). *rip3*^−/−^ mice were provided by Drs. Kim Newton and Vishva Dixit (Genentech) (Newton et al., 2004). All animal studies were approved by the Institutional Animal Care and Use Committee (IACUC) at Thomas Jefferson University.

### Mouse genetic analysis

*rip1^+/−^fadd^+/−^* mice were crossed with *rip3*^−/−^ mice. The resulting *rip1^+/−^fadd^+/−^rip3^+/−^* triple heterozygous (THZ) mice were intercrossed. From the offspring, *rip1^+/−^fadd^−/−^rip3*^−/−^ mice were mated with THZ mice, producing viable and fertile *rip1^−/−^fadd^−/−^rip3*^−/−^ triple knockout (TKO) mice, which were mated with *rip1^+/−^fadd^−/−^rip3*^−/−^ mice. For genotyping, genomic DNA was isolated from the tails of newborns to weaning-age mice using proteinase K and sodium dodecyl sulfate (SDS)-containing lysis buffer followed by phenol extraction and ethanol precipitation. Allele-specific primers were used to detect the presence of wild type and knockout alleles of *rip1* and *fadd* as previously described (Zhang et al., 2011). *rip3*^−/−^ mice were typed using the forward primers 5′-GCCTGCCCATCAGCAACT-3′ and 5′-CCAGAGGCCACTTGTGTAGCG-3′ and reverse primer 5′-CGCTTTAGAAGCCTTCAGGTTGAC-3′ (Newton et al., 2004). PCR produces a 320 bp wild type band and a 485 bp knockout band. For postnatal survival analysis, neonates were monitored for survival from birth to weaning age; deceased pups were immediately collected and genotyped. Survival curves were generated using Prism software (Graphpad Software, Inc.). For genetic analysis, the numbers of mice containing the most numbers of wild type alleles of the genes of concern were used to predict the numbers of other genotypes among the offspring of the indicated crosses, based on Mendelian ratios.

### Western blotting analysis

The presence or absence of FADD, RIP3, and RIP1 protein was confirmed by western blotting. Total splenocytes were isolated from mice of the indicated genotypes. Cell lysates were prepared in cold RIPA lysis buffer containing 50 mM Tris pH 8.0, 150 mM NaCl, 1% Nonidet P-40, 0.5% deoxycholate, 0.1% SDS, 1 mM phenylmethyl sulphonyl fluoride (PMSF), and a proteinase inhibitor cocktail (Roche). Proteins (30 μg) were separated on a 10% SDS-PAGE gel and transferred to a nitrocellulose membrane. Proteins on the membrane were stained with Ponceau *S* (Sigma-Aldrich) as a loading/transfer control. Antibodies specific for FADD (generated in house), RIP3 (ProSci, Catalog #2283), and RIP1 (BD Biosciences, Catalog #610459) were incubated with the membrane overnight at 4°C followed by HRP-conjugated goat anti-rabbit antibodies (1/10,000). Chemiluminescent signals were detected on the ProteinSimple FluorChem M Western Blot Imaging machine.

### Flow cytometric analysis

Spleen, lymph nodes, and thymus of mice were isolated during mouse dissection. Red blood cell lysis of the spleen was performed using ACK buffer and single cell suspensions were prepared. Cells (10^6^) were stained with fluorochrome-conjugated antibodies in PBS containing 3% BSA, 0.5 mM EDTA, and 0.05% sodium azide, and samples were incubated on ice for 30 min. Antibodies used for flow cytometry were anti-CD3 (BD Biosciences), anti-CD4 (Caltag), anti-CD8 (Invitrogen), and anti-B220 (BD Biosciences). Data were acquired on an LSR II cytometric analyzer (BD Biosciences) and analyzed using FlowJo software (TreeStar).

### Cell culture

Thymocytes and peripheral T cells purified from the spleen and lymph nodes were cultured in complete RPMI media (cRPMI) containing 10% FBS, 2 mM L-Glutamine, penicillin (100 U/mL), streptomycin (100 μg/mL), and β-mercaptoethanol (50 μM).

### Total cellularity calculations

Total cell number was obtained using single cell suspensions spleen and lymph nodes counted using a Countess Automated Cell Counter (Invitrogen). Cell number of various lymphocyte subsets was obtained by multiplying the total cell number with percentages of subsets of CD3^+^ and B220^+^ cells obtained by flow cytometry.

### Cell death assays

Thymocytes (10^5^) were seeded in a 96-well flat-bottom plate in 100 μL of cRPMI and incubated at 37°C with 5% CO_2_for 16 h with indicated concentrations of anti-Fas antibodies, TNFα, or dexamethasone. Thymocytes were also incubated with no treatment, staurosporine (1 μg/mL), and ionomycin (1 μg/mL) for indicated time points. Cells were collected, each well was washed with PBS, and 1 μg/mL propidium iodide was added to the sample. Cells were analyzed by flow cytometry on an LSR II cytometric analyzer (BD Biosciences). In some cases, Percent Specific Death was calculated by: (percent cell killing with treatment)—(percent cell killing without treatment).

### Proliferation assays

T cells were purified by pooling total spleen and lymph node cells of five-week-old mice and using antibodies against Thy1.2 and B220. Thy1.2^+^B220^−^ T cells were sorting using a FACS Aria (BD Biosciences). Purified T cells were labeled with CellTrace Violet (Life Technologies), as per the manufacturer's instructions. Cells were placed in a 96 well round-bottom plate (2 × 10^5^ cells/well) and stimulated with 1 μg/mL anti-CD3 antibody and 0.2 μg/mL anti-CD28 antibody in 100 μL cRPMI for various timepoints. Cells were stained with 1 μg/mL propidium iodide and run on an LSR II cytometric analyzer (BD Biosciences). For ^3^H-thymidine experiments, cells were placed in a 96 well round-bottom plate (10^5^ cells/well) and stimulated with 1 μg/mL anti-CD3 antibody and 0.2 μg/mL anti-CD28 antibody for 40 h. Two microcuries of tridiated thymidine were added to each well and cells were incubated for an additional 8 h. Samples were placed onto a glass fiber filter using a Mach Harvester 96 (Tomtec) and analyzed using a Wallac 1205 Betaplate Counter (Perkin Elmer).

### Statistical analysis

Data is expressed as mean ± standard deviation. Student's *t*-tests were performed using Prism software (Graphpad Software, Inc).

## Results

### Postnatal development of *rip1*^−/−^ mice in the absence of RIP3 and/or FADD

**Figure 1:**
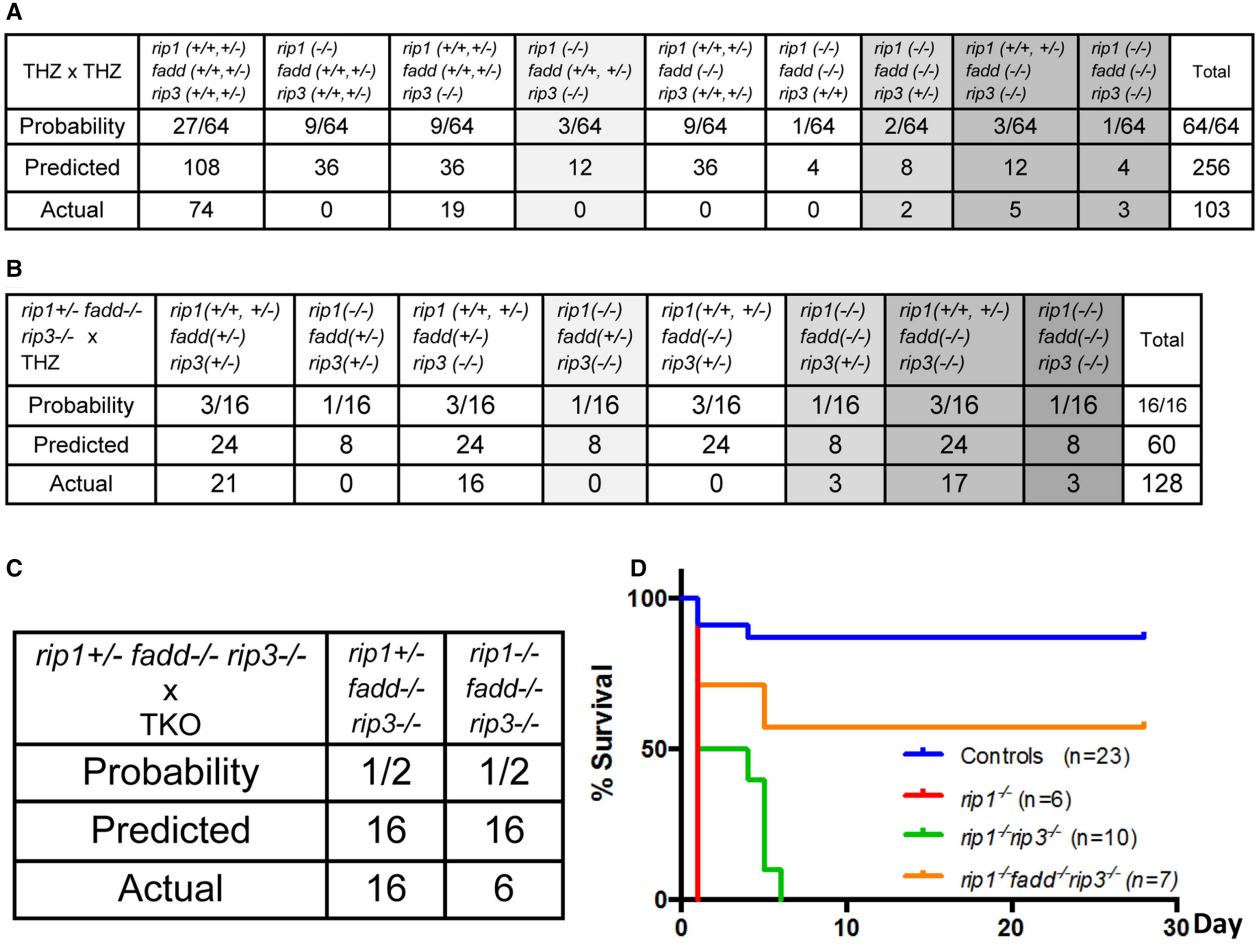
***rip1*^−/−^ perinatal lethality is mediated by FADD and RIP3. (A–C)** Predicted and actual frequency of indicated genotypes of weaning age mice resulting from intercrosses of *rip1^+/−^fadd^+/−^rip3^+/−^* THZ mice **(A)**, crosses of *rip1^+/−^fadd^+/−^rip3^+/−^* and *rip1^+/−^fadd^−/−^rip3*^−/−^ mice **(B)**, and crosses of *rip1^+/−^fadd^−/−^rip3*^−/−^ and *rip1^−/−^fadd^−/−^rip3*^−/−^ mice **(C)**. **(D)** Survival of newborn mice from various intercrosses. For this and following figures, control mice are littermates that have at least one wild type allele of FADD and RIP1. The *fadd^−/−^rip3*^−/−^ mice shown contain at least one wild type allele of *rip1*.

To determine whether RIP3 plays a role in the postnatal defect of *rip1*^−/−^ mice, *rip1^+/−^rip3^+/−^* or *rip1^+/−^rip3*^−/−^ mice were intercrossed, which produced no viable *rip1^−/−^rip3*^−/−^ double knockout (DKO) or *rip1^−/−^rip3^+/−^* mice at weaning age (data not shown). This observation was confirmed when *rip1^+/−^fadd^+/−^rip3^+/−^* triple heterozygous (THZ) mutant mice were intercrossed, which produced no viable *rip1^−/−^rip3*^−/−^ mice containing one or both alleles of FADD, though Mendelian genetics predicts 12 mice of such genotypes (column 5, Figure 1A). Nonetheless, *rip1^−/−^rip3*^−/−^ newborns were detected, and showed slightly improved survival up to postpartum day (p)6, as compared to *rip1*^−/−^ newborns which died at p1 (Figure 1D). In contrast, if one or both alleles of the *rip1* gene were present, *fadd^−/−^rip3*^−/−^ DKO mice were viable from birth to adulthood (column 9, Figure 1A).

**Figure 2:**
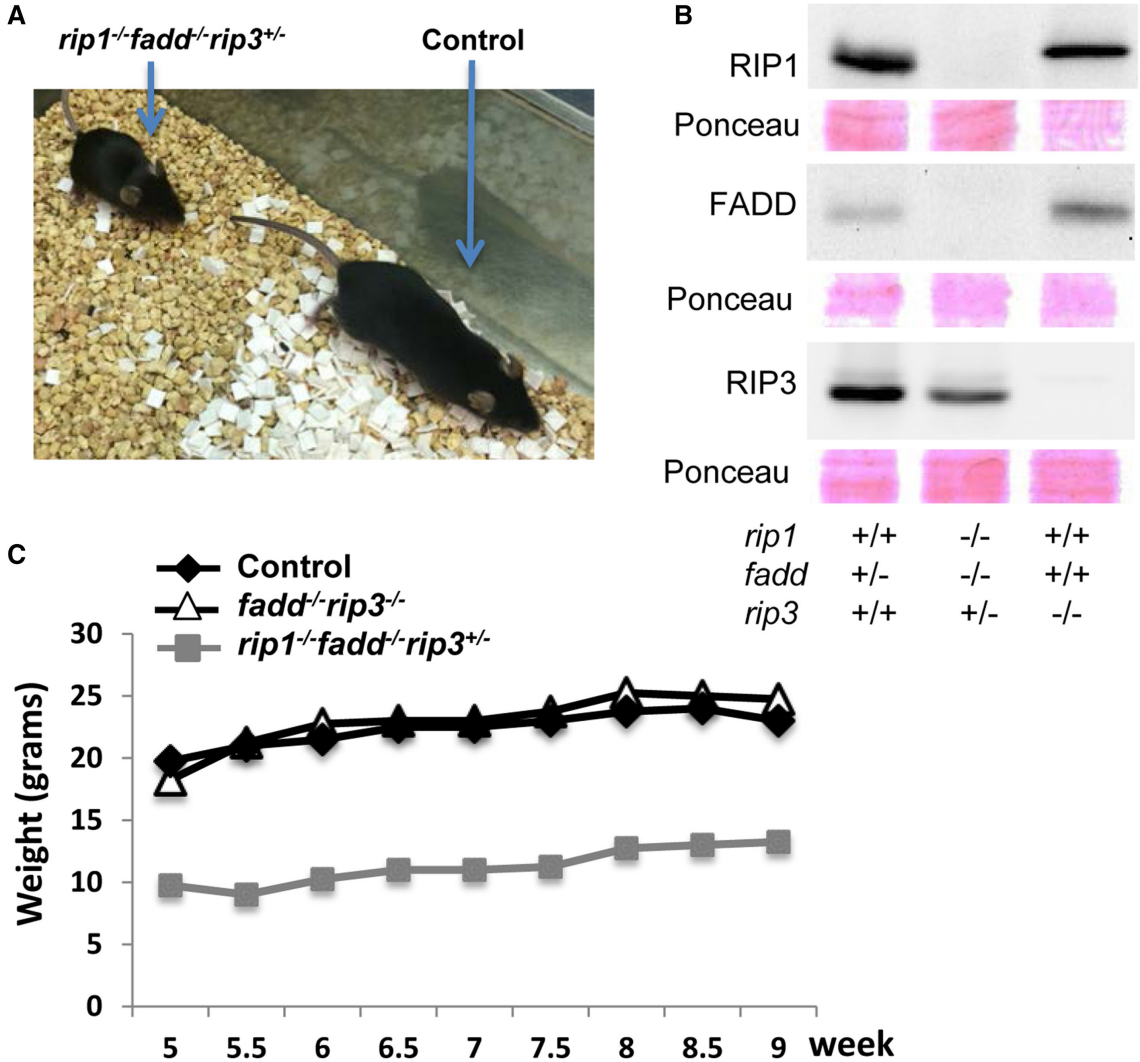
**Haploid insufficiency of *rip3* improves survival of *rip1*^−/−^*fadd*^−/−^ mice, which are viable but weigh less than wild type controls. (A)** Adult *rip1^−/−^fadd^−/−^rip3^+/−^* mouse and littermate control. **(B)** Western blot analysis of total splenocytes confirming presence or absence of FADD, RIP3, and RIP1 protein. **(C)** Weight curves showing the *rip1^−/−^fadd^−/−^rip3^+/−^* mouse with age-matched controls. Data shown is representative of three mice of each genotype.

Similar to the previous study (Zhang et al., 2011), no viable *rip1*^−/−^*fadd*^−/−^ mice were present at weaning age among the offspring of THZ intercrosses (column 7, Figure 1A). Strikingly, however, absence of one allele of RIP3 allowed survival of *rip1*^−/−^*fadd*^−/−^ mice beyond weaning ages up to adulthood (column 8, Figures 1A, 2A). Presence or absence of the FADD, RIP1, and RIP3 proteins was analyzed by western blotting (Figure 2B). These observations were also confirmed by analysis of the offspring from crosses of *rip1^+/−^fadd^−/−^rip3*^−/−^ mice with *rip1^+/−^fadd^+/−^rip3^+/−^* THZ mice (Figure 1B), which produce higher ratios of *rip1^−/−^fadd^−/−^rip3^+/−^* mice.

Further examination revealed that although *rip1^−/−^fadd^−/−^rip3^+/−^* mice were viable, they were much smaller in size (Figure 2A), and also weighed less than wild type controls or *fadd^−/−^rip3*^−/−^ mice during the indicated period of analysis (Figure 2C). Moreover, *rip1^−/−^fadd^−/−^rip3^+/−^* mice appeared to be present at lower numbers (2) at weaning age than the predicted Mendelian frequencies (8) (column 8, Figure 1A). Western blotting analysis indicated that ablation of one allele of RIP3 resulted in lower RIP3 protein levels (Figure 2B), which helped improve survival of *rip1*^−/−^*fadd*^−/−^ mice.

**Figure 3:**
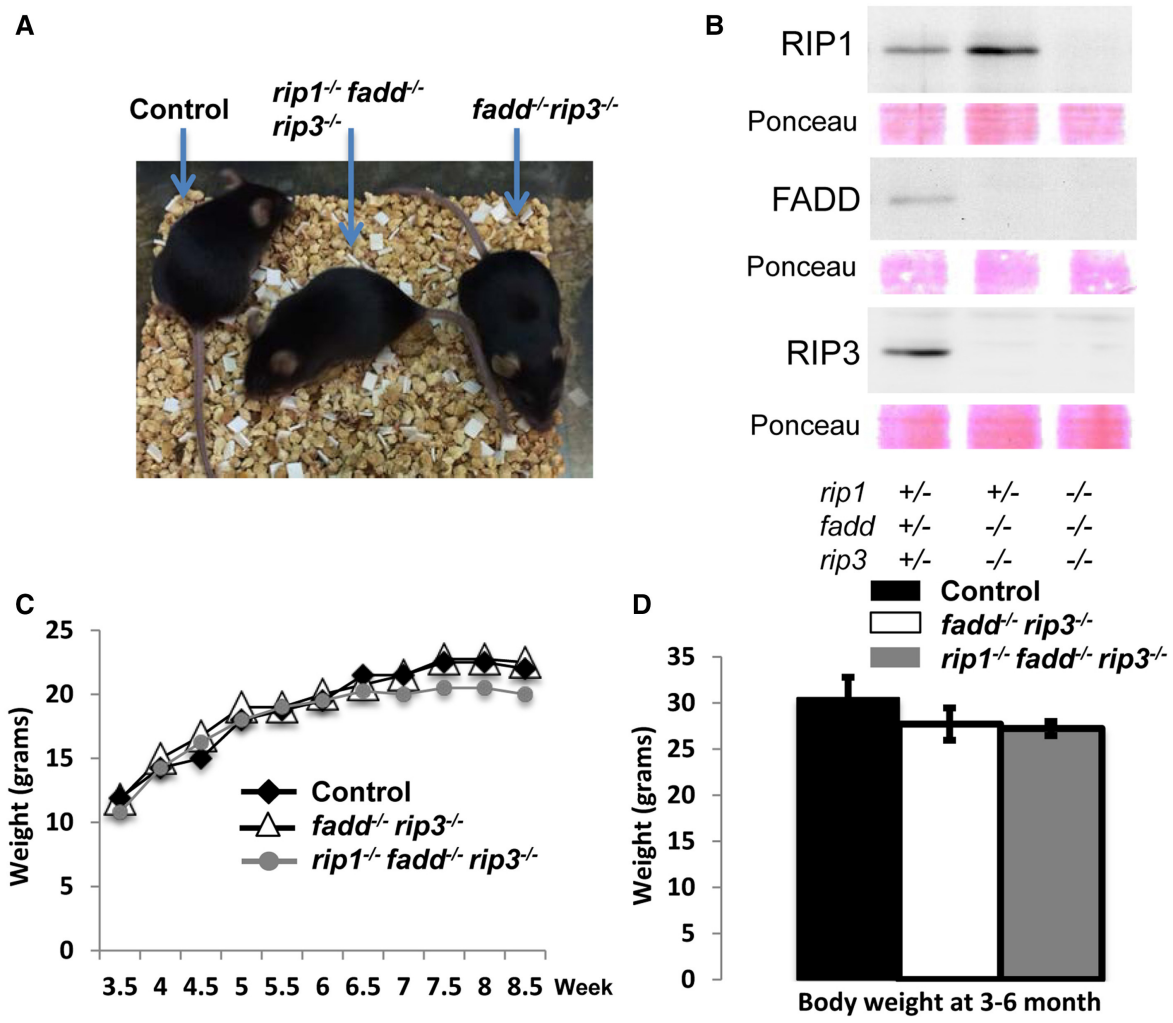
**Complete ablation of both RIP3 and FADD rescued *rip1*^−/−^ perinatal lethality. (A)** *rip1^−/−^fadd^−/−^rip3*^−/−^ mouse with *fadd^−/−^rip3*^−/−^ and littermate control. **(B)** Western blot of total splenocytes confirming presence or absence of FADD, RIP3, and RIP1 protein. **(C)** Weight curves of *rip1^−/−^fadd^−/−^rip3*^−/−^ with age-matched controls, representative of at least three mice of each genotype. **(D)** Body weight of 3- to 6-month-old mice at time of analysis. Data shown is representative of four mice of each genotype.

Interestingly, when both alleles of RIP3 were absent, the resulting *rip1^−/−^fadd^−/−^rip3*^−/−^ triple knockout (TKO) mice gained weight normally, as compared to wild type control or *fadd^−/−^rip3*^−/−^ DKO mice (Figures 1, 3A,C,D). Nonetheless, the numbers of the TKO mice appeared to be lower than that predicted by Mendelian ratios, particularly among the offspring of the mouse cross in Figure 1B (column 10). This defect is more pronounced through analyzing the offspring of crosses between *rip1^+/−^fadd^−/−^rip3*^−/−^ mice and TKO mice (Figure 1C). This correlates with decreased survival of *rip1^−/−^fadd^−/−^rip3*^−/−^mice from birth to weaning age (Figure 1D). Knockout of these genes was confirmed by western blotting (Figure 3B). In total, these data showed that while *rip1^−/−^rip3*^−/−^ mice did survive several days longer than *rip1*^−/−^, knockout of both FADD and RIP3 was necessary for survival of *rip1*^−/−^ mice to adulthood.

### The role of RIP1, FADD, and RIP3 in lymphocyte responses

**Figure 4:**
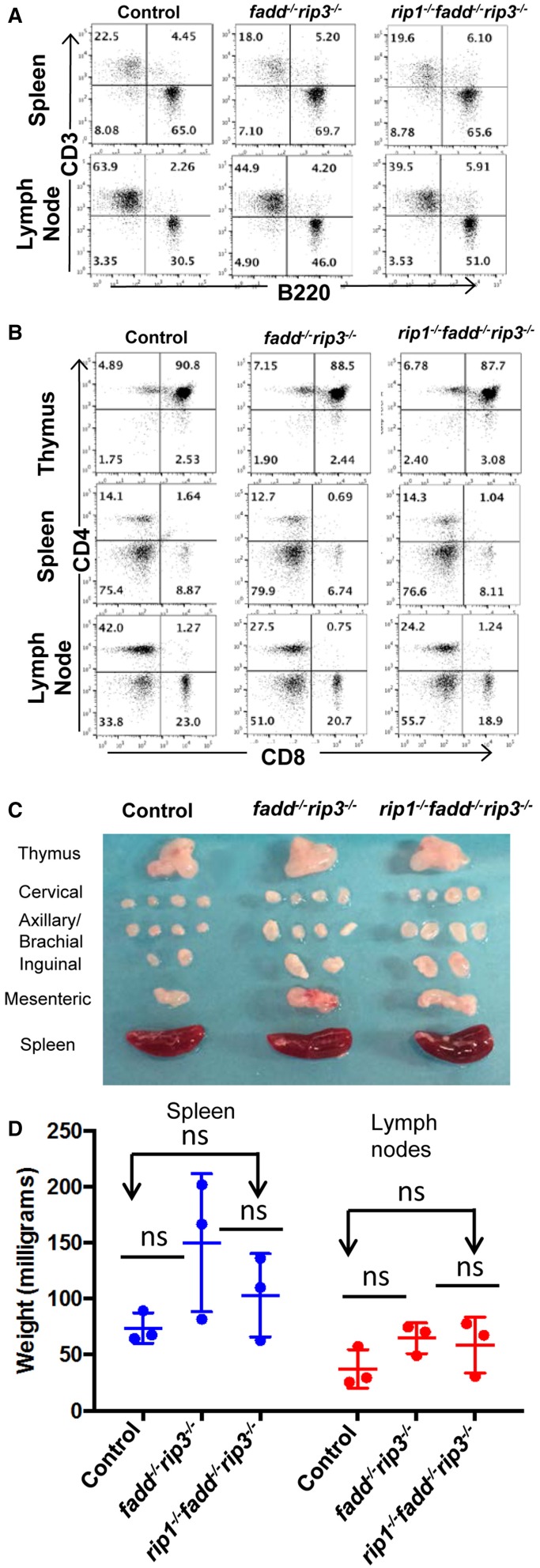
**Young *rip1^−/−^fadd^−/−^rip3*^−/−^ mice contain normal T and B cell populations. (A,B)** Spleen, lymph node, and thymus of 5 week old mice of the indicated genotypes were stained with antibodies for CD3 and B220 **(A)** or CD4 and CD8 **(B)**. Numbers indicated are percentages of T cell and B cell subsets. **(C)** Lymph nodes, spleen, thymus of 5 week-old mice. **(D)** Weight of spleen and lymph node of mice 4–6 week old mice. ns, not significant. Data shown is representative of at least three independent experiments with one mouse of each genotype.

The role of these proteins in lymphocyte development and function has been previously investigated through gene deletion in germ cells or in specific lineages. The lymphoid system of *rip3*^−/−^ mice is normal (Newton et al., 2004). Without FADD function, peripheral T cell number decreases (Walsh et al., 1998; Zhang et al., 2005), but the B cell compartment is normal (Imtiyaz et al., 2006). Lastly, inducible deletion of RIP1 results in greatly reduced lymphocyte numbers (Roderick et al., 2014). We initially analyzed young (4 week old) mice, due to concerns of age-dependent diseases (see below). The *fadd^−/−^rip3*^−/−^ DKO and *rip1^−/−^fadd^−/−^rip3*^−/−^ TKO mutant mice contained normal T cell populations in the thymus, similar to that in control mice (Figure 4B). This indicates that RIP1 deficiency does not affect T lymphocyte development, as long as FADD and RIP3 are absent. The lymphoid organs of DKO and TKO mice at 5 weeks of age were not significantly larger than control mice, and these mutant mice appeared to contain higher numbers of B cells in the lymph nodes (Figures 4A,C,D).

**Figure 5:**
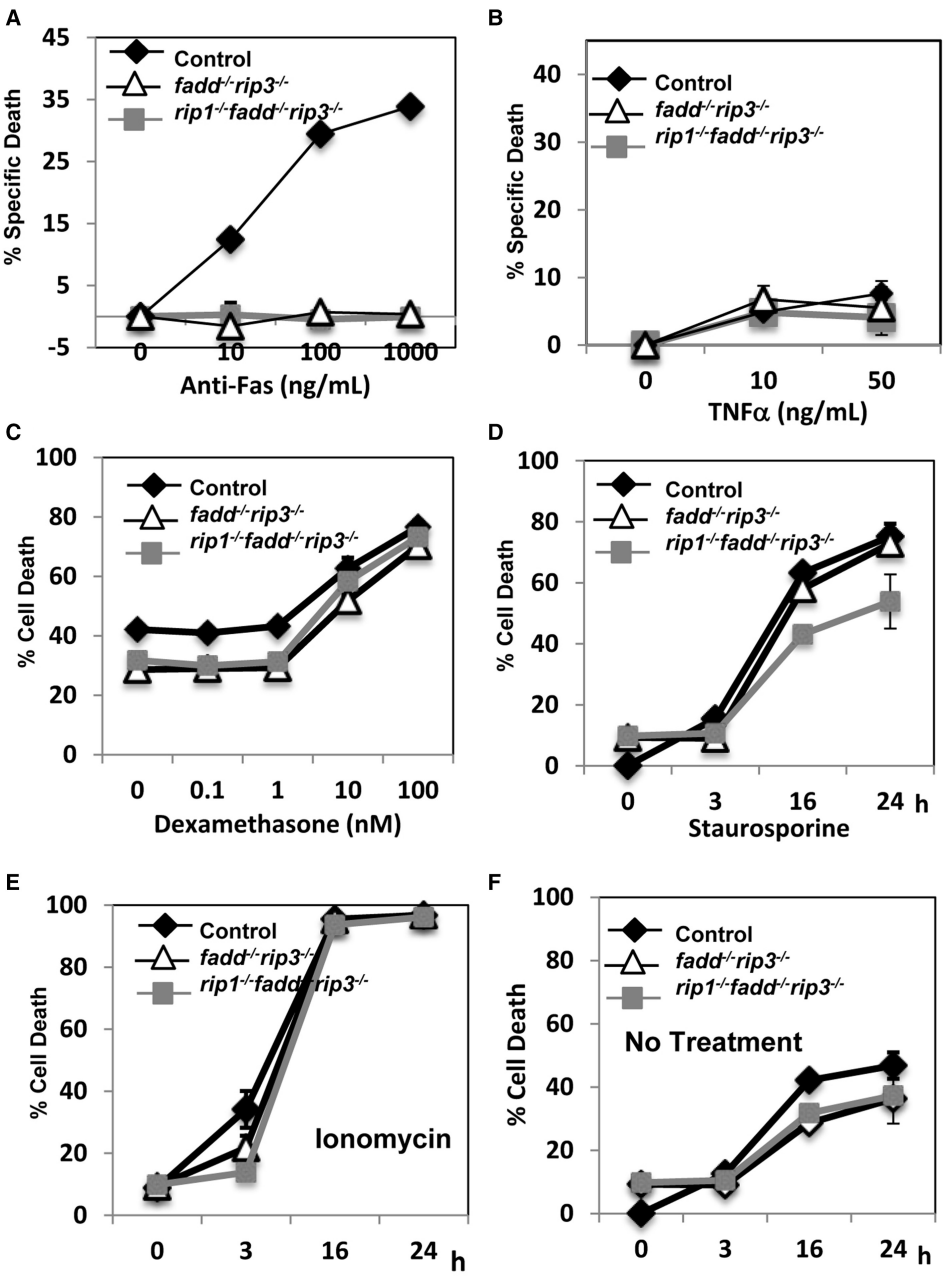
***rip1^−/−^fadd^−/−^rip3*^−/−^ mice had impairment of extrinsic, but not intrinsic, cell death pathways. (A–D)** Thymocytes of indicated genotypes were treated with various doses of **(A)** anti-Fas antibody, **(B)** TNFα, **(C)** Dexamethasone and cell death was determined by PI uptake after 16 h incubation. **(D–F)** Thymocytes were treated with **(D)** staurosporin (1 μg/ml), **(E)** ionomycin (1 μg/mL) or **(F)** no treatment for indicated time and cell death was determined by PI uptake. Experiments were performed in triplicate.

The extrinsic cell death pathways mediated by the DRs are essential for immune homeostasis, as indicated by the *lpr* mouse model. Disruption of FADD function blocks Fas-induced apoptosis in T cells (Walsh, 2014). We previously have shown that absence of RIP1 has no effect on Fas-induced death, but leads to hypersensitivity to TNF-induced death in thymocytes (Zhang et al., 2011). In contrast, *rip1*^−/−^*fadd*^−/−^ thymocytes were resistant to TNF, similar to wild type thymocytes (Zhang et al., 2011). In order to determine the status of these pathways in *rip1^−/−^fadd^−/−^rip3*^−/−^ mice, thymocytes were isolated and treated with various stimuli to initiate extrinsic or intrinsic cell death. Similar to *fadd^−/−^rip3*^−/−^ DKO thymocytes, TKO thymocytes were highly resistant to cell death responses induced by Fas (Figure 5A). Unlike *rip1*^−/−^ thymocytes (Zhang et al., 2011), TKO and DKO mutant thymocytes were not sensitive to TNFα-induced cell death (Figure 5B). However, they were able to undergo intrinsic apoptosis in response to dexamethasone, similar to wild type thymocytes (Figure 5C). Furthermore, DKO and TKO thymocytes were sensitive to staurosporine and ionomycin-induced intrinsic apoptosis, as compared with untreated cells (Figures 5D–F). These results imply that resistance to extrinsic cell death is essential for the survival of *rip1*^−/−^ mice.

**Figure 6:**
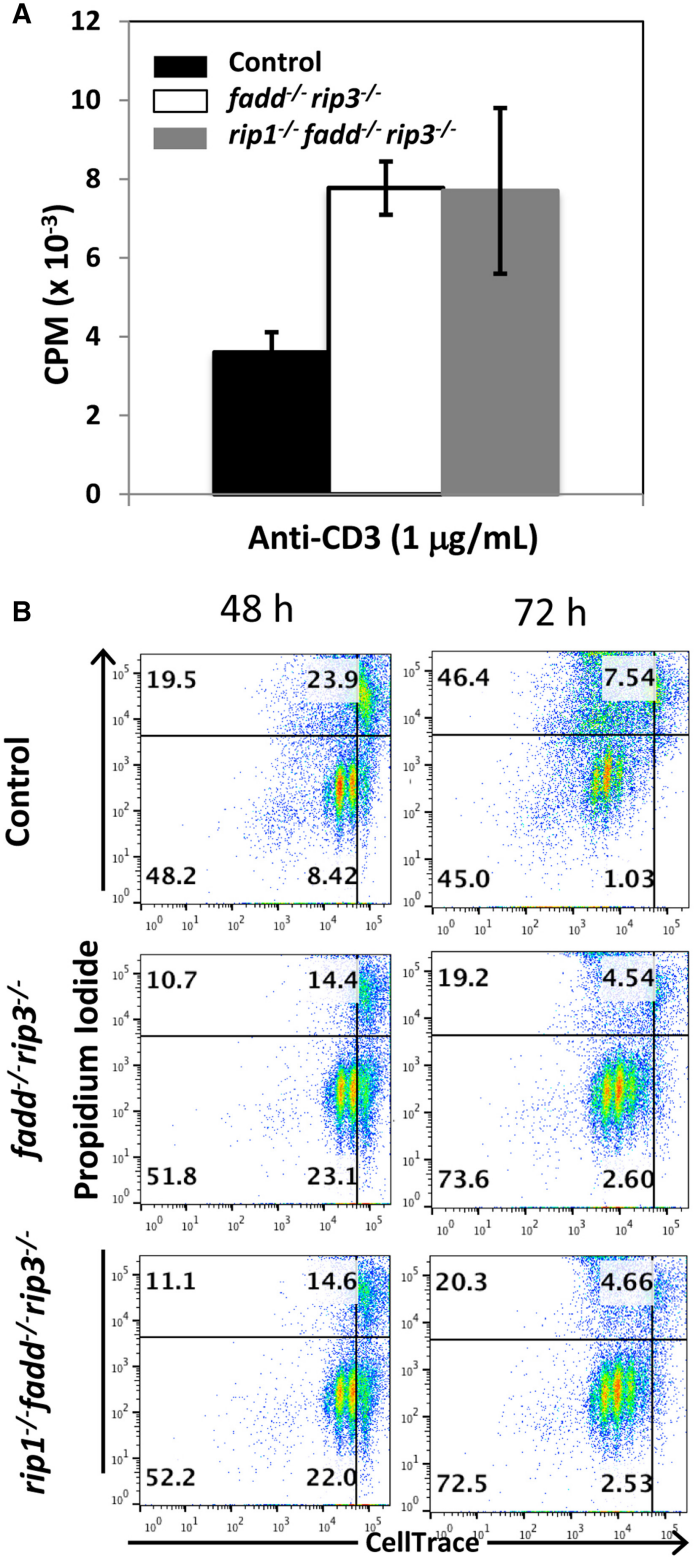
**Proliferative responses of TKO and DKO T cells are more robust than control T cells. (A)** T cells of indicated genotypes were stimulated with anti-CD3, anti-CD28 antibodies. Proliferation was measured by [^3^H]-thymidine incorporation (cpm, counts per minute). **(B)** T cells labeled with CellTrace dye were stimulated with anti-CD3, anti-CD28 antibodies. Dilution of CellTrace dye and uptake of propidium iodide was monitored by flow cytometry at various time points. Data shown is representative of at least three independent experiments with one mouse of each genotype.

Previous studies indicated that FADD is required during lymphocyte proliferative responses, to sequester RIP1/RIP3-dependent necrosis (Lu et al., 2011; Zhang et al., 2011). To analyze TCR-induced proliferation, peripheral T cells were isolated and stimulated with anti-CD3 antibodies. In tritiated thymidine incorporation assays, there appeared to be more proliferation in *fadd^−/−^rip3*^−/−^ T cells and *rip1^−/−^fadd^−/−^rip3*^−/−^ T cells than control T cells (Figure 6A). In further analysis, T cell division kinetics and death responses were analyzed through two-color flow cytometry. T cells were pre-labeled with the CellTrace dye, and then stimulated with anti-CD3/CD28 antibodies. T cell division is indicated by dilution of CellTrace, and cell death measured through propidium iodide dye staining. As shown in Figure 6B, accelerated proliferative responses were seen in both *fadd^−/−^rip3*^−/−^ and *rip1^−/−^fadd^−/−^rip3*^−/−^ T cells, when compared to control T cells. Moreover, there was less cell death in DKO and TKO mutant T cells than in control T cells (Figure 6B).

### The role of RIP1 in age-dependent *lpr* diseases

**Figure 7:**
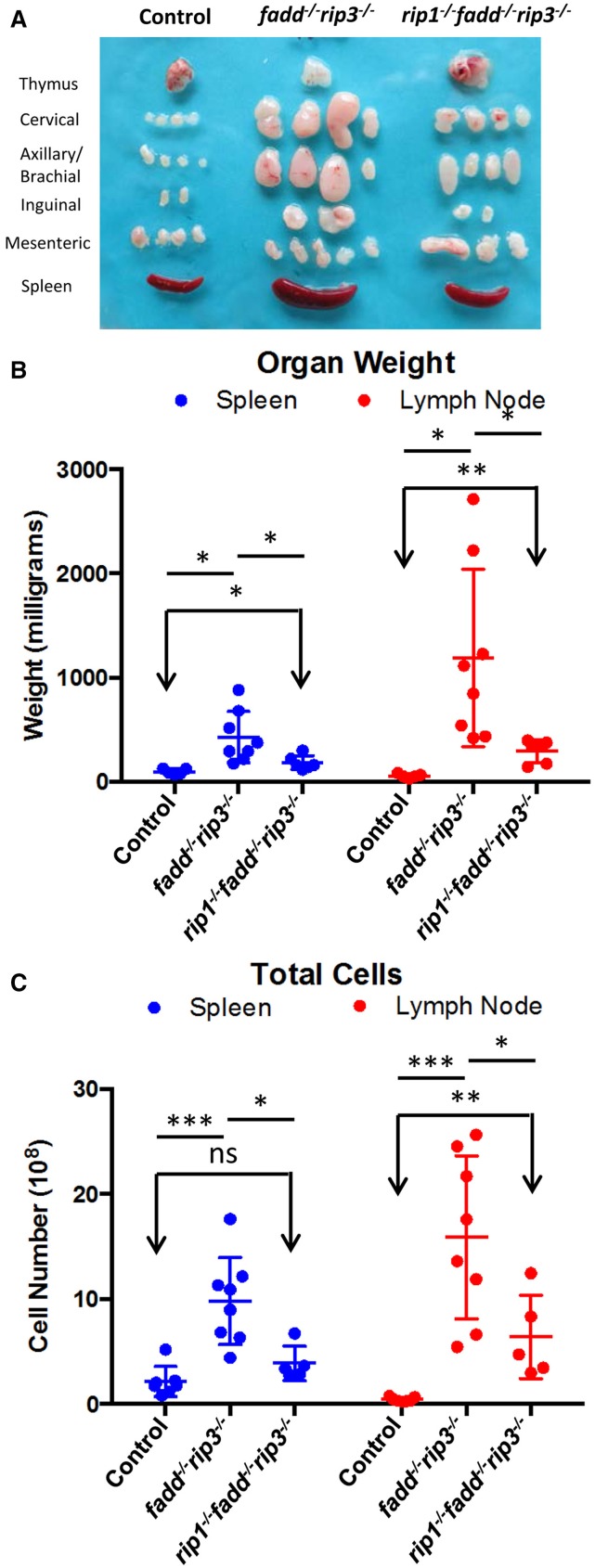
**Lymphadenopathy and splenomegaly is less severe in TKO mice, compared to DKO mice**. **(A)** Thymus, lymph nodes, and spleen of 6-month-old mice **(B)** Weight of spleen and lymph nodes of mice >3 months old **(C)** Total cell number of spleen and lymph node of mice 3–6.5 months old. At least five mice from each group were used to generate given data. ^*^p < 0.05; ^**^p < 0.01; ^***^p < 0.001; ns, not significant.

Previous studies have shown that disruption of the function of FADD by using a FADD-DN mutant results in an *lpr*-like symptom in aged *rip3*^−/−^ mice (Lu et al., 2011). Similarly, *fadd^−/−^rip3*^−/−^ DKO mutant mice developed *lpr*-like symptoms including enlarged lymph nodes and spleen in as early as 5-week old mice (Figure 7 and data not shown). As mice age, the lymphadenopathy and splenomegaly phenotype become more pronounced, as indicated by greatly increased size, weight, and total cellularity of the secondary lymphoid organs of the *fadd^−/−^rip3*^−/−^ DKO mice (Figures 7A–C). Interestingly, these signature *lpr* symptoms were diminished in TKO mice, as compared to DKO mice (Figure 7).

**Figure 8:**
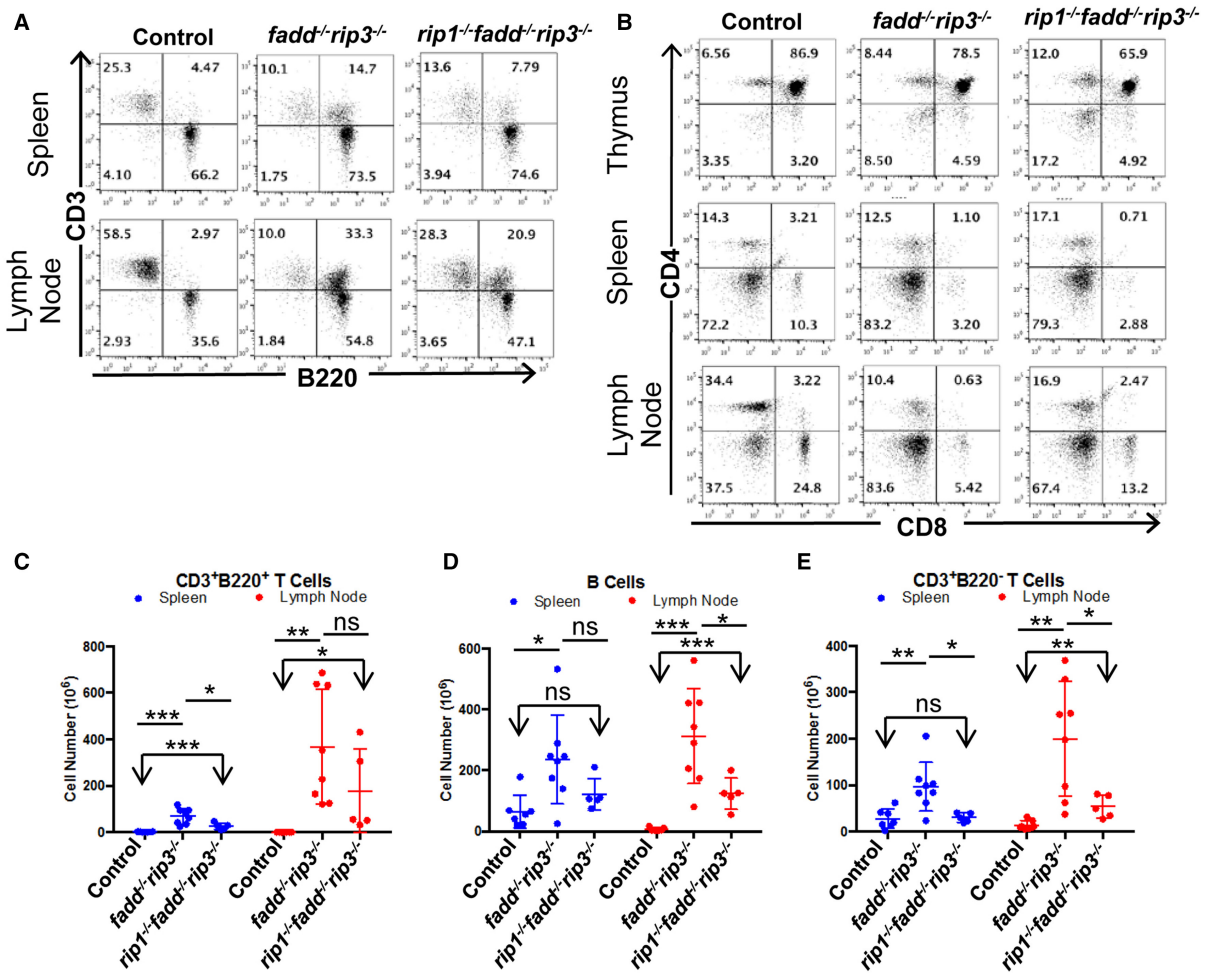
**Knockout of RIP1 reduced severity of the *lpr* symptoms of lymphadenopathy and splenomegaly in *fadd^−/−^rip3*^−/−^**. **(A,B)** Spleen, lymph node, and thymus of six-month-old mice of the indicated genotypes were stained with antibodies for CD3 and B220 **(A)** or for CD4 and CD8 **(B)**. Numbers indicated are percentages of T cell and B cell subsets. **(C–E)** Scatter plots of total number of **(C)** CD3^+^B220^+^ T Cells, **(D)** B cells, and **(E)** conventional CD3^+^B220^−^ T cells. At least five mice from each group were used to generate given data. ^*^p < 0.05; ^**^p < 0.01; ^***^p < 0.001; ns, not significant.

Flow cytometric analyses showed that DKO mice contained the unique subset of CD3^+^ T cells, which express the B cell marker B220^+^, a signature phenotype of the Fas mutant mice (Figure 8A). Furthermore, these T cells lose the expression of CD4 and CD8 (Figure 8B). When absolute numbers of distinct lymphocyte populations were determined, DKO mice aged 3–6 months were shown to accumulate dramatically higher numbers of CD3^+^B220^+^ T cells (Figure 8C). Moreover, B cells and conventional T cells (CD3^+^B220^−^) number also increased in DKO mice (Figures 8D,E). Such *lpr*-like phenotypes were significantly less severe when RIP1 was deleted (Figures 7, 8).

## Discussion

PCD is tightly controlled in order to ensure proper embryonic and postnatal development as well as to maintain tissue homeostasis. Two major PCD pathways, intrinsic and extrinsic apoptosis, have been well-studied. Intrinsic apoptosis is mediated by pro-apoptotic members of the Bcl-2 family proteins including Bax and Bak, which are counteracted by Bcl-2 and Bcl-X_S/L_ (Marsden and Strasser, 2003; Danial and Korsmeyer, 2004). Absence of Bcl-X_S/L_ blocks embryogenesis at early-midgestation stages due to massive cell death, and deletion of Bcl-2 results in lymphopenic conditions due to greatly increased apoptosis in lymphocytes. In contrast, when proapoptotic Bim is inactivated, mice develop autoimmune and lymphoproliferative diseases (Bouillet et al., 2002). The significance of extrinsic PCD pathways during animal development mediated by DRs or TLRs has been underappreciated, mainly because mice lacking these receptors undergo normal embryonic and postnatal development. As such, developmental defects in mice without the downstream signaling proteins of the DRs, including RIP1, FADD, Caspase 8, or cFLIP, remained largely a conundrum for over a decade, until a latent, RIP1/RIP3-dependent necrosis-like death pathway has been revealed recently (Moriwaki and Chan, 2013; Walsh, 2014).

The fact that RIP1, as implicated in studies based on cell systems, is involved in the regulation of NF-κB activation, apoptosis, and necroptosis, poses as a challenge in understanding the perinatal lethality phenotype of *rip1*^−/−^ mice (Kelliher et al., 1998). One possible mechanism is that *rip1*^−/−^ cells become hypersensitive to extrinsic cell death, as observed previously(Ting et al., 1996; Kelliher et al., 1998). However, our previous study shows that blocking extrinsic apoptosis pathways alone through deletion of FADD is insufficient, as postnatal survival of *rip1*^−/−^*fadd*^−/−^ mice were only marginally improved (Zhang et al., 2011). Alternatively, evidence exists, which implies that RIP3 mediates necroptosis in the absence of RIP1 (Upton et al., 2010; Moujalled et al., 2013). Indeed, we found that deletion of RIP3 helped improve the survival of *rip1*^−/−^ mice for several additional days (Figure 1 and data not shown). Importantly, the current study demonstrates that postnatal lethality in *rip1*^−/−^ mice is due to the functions of not only FADD but also RIP3, because *rip1*^−/−^ mice could reach adulthood only if both the FADD and RIP3 genes are ablated simultaneously (Figures 1–3). Firstly, decreasing the RIP3 protein level through deletion of just one allele of the RIP3 gene significantly improved survival of *rip1*^−/−^*fadd*^−/−^, although the resulting *rip1^−/−^fadd^−/−^rip3^+/−^* mice had trouble gaining weight (Figures 1, 2). This could suggest that the initial pathology of cell death in the adipose tissue *rip1*^−/−^ mice is partially mediated by RIP3, which may explain the systemic inflammation displayed in *rip1^−/−^fadd^−/−^rip3^+/−^* mice, as indicated in a previous independent study (Kaiser et al., 2014). Secondly, when both alleles of RIP3 were deleted, the resulting TKO mice show no defect in body sizes and weights as compared to DKO or control littermates (Figure 3). This data implies that during postnatal development, *rip1*^−/−^ cells may undergo both FADD-mediated apoptosis and RIP3-dependent necroptosis. This new finding is counterintuitive because RIP1 appears to signal through RIP3 in promoting necroptosis in embryonic cells lacking FADD or Caspase 8 or cFLIP (Kaiser et al., 2011; Oberst et al., 2011; Dillon et al., 2012). In particular, our previous study showed that deletion of the RIP1 adaptor kinase is able to correct the embryonic defect in *fadd*^−/−^ mice (Zhang et al., 2011). Moreover, *fadd*^−/−^ or *caspase 8*^−/−^ mice could reach adulthood when RIP3 is deleted (Kaiser et al., 2011; Oberst et al., 2011; Dillon et al., 2012). It appears that RIP1 plays distinct roles at different stages during development. While dispensable in embryos, RIP1 is required to restrict FADD-mediated apoptosis and RIP3-dependent necroptosis in order to ensure proper postnatal development.

The precise mechanism involved in RIP1-mediated sequestration of apoptosis and necroptosis remains to be further defined. One scenario involves cFLIP, an inhibitor of both apoptosis and necroptosis. cFLIP is a target of NF-κB (Kreuz et al., 2001), which could be activated through RIP1 under certain circumstances. Likely, lack of RIP1 impairs the expression of cFLIP. It was previously thought that the defect in *rip1*^−/−^ mice is due, in part, to TNFR1-triggered apoptosis. Recently, deletion of TNFR1 or caspase 8 in addition to RIP1 was attempted to mitigate uncontrolled apoptosis, but provided no survival advantage (Rickard et al., 2014). A RIP1-independent necroptotic pathway is induced by TLR3 and TLR4 through its adaptor TIR-domain-containing adaptor-inducing interferon-β (TRIF), which can bind RIP3 via the RIP homotypic interaction motif (RHIM) (Kaiser et al., 2013; Dillon et al., 2014). These two TLRs could also trigger activation of NF-κB through RIP1 (Meylan et al., 2004; Zhang et al., 2011). The type I and type II interferon (IFN) receptors are also capable of inducing necroptosis (Balachandran et al., 2004; Kaiser et al., 2014). In concordance with these models, knockout of TRIF or the type I IFN receptor, in addition to knockout of RIP1 and TNFR, modestly prolonged survival, allowing a small number of these mice to survive to weaning age (Dillon et al., 2014). The perinatal lethality of *rip1*^−/−^ mice was partially rescued when apoptosis or necroptosis mediated by TNFR, TLRs, or IFN receptors was blunted (Dillon et al., 2014; Kaiser et al., 2014; Rickard et al., 2014).

RIP3 and MLKL have been shown to execute necroptosis in a RIP1-independent manner through Toll-like Receptor-3 (TLR-3) (Upton et al., 2010; He et al., 2011; Kaiser et al., 2013). Recent independent study has shown that ablation of either RIP3 or MLKL in addition to RIP1 resulted in lower levels of cleaved caspase 3 in various organs in newborns compared with *rip1*^−/−^ newborns as well as lower levels of inflammatory cytokines, potentially due to lower levels of damage-associated molecular patterns (DAMPs) released from necrotic cells (Rickard et al., 2014). However, these mice did not survive past day p4. Another study found that a small percentage of *rip1^−/−^rip3*^−/−^ mice survived up to 21 days, however, we see no survival of *rip1^−/−^rip3*^−/−^ mice beyond day p6 (Figure 1D). Furthermore, some *rip1^−/−^tnfr*^−/−^ survived for 18 days (Dillon et al., 2014), whereas such mice die right at birth in a separate study (Rickard et al., 2014). Combined knockout of RIP3 and TNFR1 did allow survival of *rip1*^−/−^ mice into adulthood, but these mice ultimately succumbed to blood bacteremia, sepsis, and prevalent apoptosis in the intestines (Dillon et al., 2014). This lethal intestinal defect caused by RIP1 ablation that was recently characterized using cell-specific Cre-mediated deletion of RIP1 (Dannappel et al., 2014; Takahashi et al., 2014). In another study, it was noted that *rip1^−/−^rip3^−/−^tnf*^−/−^ mice did not survive past day 28 (Kaiser et al., 2014). The discrepancy in survival of mice in these studies could be due to differences in genetic backgrounds of the mice used. The lethality of *rip1^−/−^rip3^−/−^tnfr*^−/−^ mice also suggests a key role for FADD/caspase 8-mediated apoptosis in RIP1 perinatal lethality. Therefore, lethality of RIP1 knockout mice is due to multiple factors.

Thymocytes isolated from DKO and TKO mice were resistant to death induced by the extrinsic pathways triggered through stimulations with anti-Fas antibodies or TNFα (Figures 5A,B). However, these mutant T cells were sensitive to cell death induced by dexamethasone, staurosporine, or ionomycin, similar to that of the wild-type control mice (Figures 5C,D,E). Extrinsic cell death has been shown to play a key role in lymphocyte homeostasis and peripheral tolerance (Strasser et al., 2009). When lymphocytes are not capable of being eliminated in the periphery, progressive accumulation of lymphocytes can occur in the peripheral lymphoid organs. This is the case in *lpr* mice, displaying splenomegaly and lymphadenopathy as a result of a mutation in the Fas gene. Whereas disruption of the FADD function alone through gene deletion or using FADD-DN results in a lymphopenic condition (Newton et al., 1998; Walsh et al., 1998; Zhang et al., 2005), double deletion of both FADD and RIP3 leads to *lpr*-like symptoms (Figures 7, 8), which is similar to that in *caspase8^−/−^rip3*^−/−^ mice (Kaiser et al., 2011; Oberst et al., 2011), but is more severe that in *fas*^−/−^ mutant mice. Notably, however, a diminished *lpr* disease was present in *rip1^−/−^fadd^−/−^rip3*^−/−^ mice (Figures 7, 8). This indicates that RIP1 has additional functions besides blocking FADD-mediated apoptosis and RIP3-dependent necroptosis. Interestingly, *rip1^−/−^fadd^−/−^rip3^+/−^* mice develop splenomegaly, but not lymphadenopathy, after several months of age (data not shown), indicating a role for RIP3-dependent necroptosis in the clearance of lymphocytes in the lymph nodes, but not the spleen. These *rip1^−/−^fadd^−/−^rip3^+/−^*, as well as DKO and TKO mice, contain varying numbers of the peculiar CD3^+^B220^+^ T cells, which lose CD4 and CD8 expression (Figure 8 and data not shown). These data, in total, illustrate that in the immune system, chronic activation of lymphocytes disrupts homeostasis and leads to autoimmunity, which can be prevented by FADD-mediated apoptosis, a sterile PCD. RIP3-deficient T cells undergo normal activation-induced cell death (AICD), indicating that FADD-mediated apoptosis is sufficient for maintaining homeostasis. Intuitively, apoptosis is a preferred pathway for AICD as necroptosis may potentially lead to inflammation which needs to be suppressed by FADD. Therefore, although pro-inflammatory, RIP1/RIP3-dependent necroptosis serves as a backup, when the FADD pathway is compromised. It is interesting to note that there appeared to be more cell division in activated *fadd^−/−^rip3*^−/−^ T cells than in wild type T cells (Figure 6). This may due in part to decreased cell death in activated *fadd^−/−^rip3*^−/−^ T cells, and/or a role for FADD in negatively regulating T cell proliferative responses. As such, both of these functions of FADD likely contribute to the greatly accelerated *lpr* disease in *fadd^−/−^rip3*^−/−^ mice.

Clearly, RIP1 plays an essential role in the life of an organism and the absence of this protein can only be compensated for by removing both apoptosis and necroptosis pathways. Due to its essential function of regulating not only cell death, but also cell survival, there may be further important consequences for the loss of such a key protein. Future investigations will drive the discoveries of the essential functions of RIP1 and the consequences for deleting RIP1. These studies will broaden our understanding of RIP1 function and could lead to the development of new strategies to target inflammatory conditions as well as cancer and autoimmune diseases, that may be exacerbated due to unregulated necrosis.

### Conflict of interest statement

The authors declare that the research was conducted in the absence of any commercial or financial relationships that could be construed as a potential conflict of interest.
